# Quick Discrimination of A_delta_ and C Fiber Mediated Pain Based on Three Verbal Descriptors

**DOI:** 10.1371/journal.pone.0012944

**Published:** 2010-09-23

**Authors:** Florian Beissner, Amadeus Brandau, Christian Henke, Lisa Felden, Ulf Baumgärtner, Rolf-Detlef Treede, Bruno G. Oertel, Jörn Lötsch

**Affiliations:** 1 pharmazentrum frankfurt/The Center for Drug Research, Development and Safety (ZAFES), Institute of Clinical Pharmacology, Goethe-University, Frankfurt am Main, Germany; 2 Clinic of Neurology, Goethe-University, Frankfurt am Main, Germany; 3 Division of Neurophysiology, Center of Biomedicine and Medical Technology Mannheim (CBTM), Medical Faculty Mannheim, Ruprecht-Karls-University Heidelberg, Mannheim, Germany; Tokyo Institute of Psychiatry, Japan

## Abstract

**Background:**

A_δ_ and C fibers are the major pain-conducting nerve fibers, activate only partly the same brain areas, and are differently involved in pain syndromes. Whether a stimulus excites predominantly A_δ_ or C fibers is a commonly asked question in basic pain research but a quick test was lacking so far.

**Methodology/Principal Findings:**

Of 77 verbal descriptors of pain sensations, “pricking”, “dull” and “pressing” distinguished best (95% cases correctly) between A_δ_ fiber mediated (punctate pressure produced by means of von Frey hairs) and C fiber mediated (blunt pressure) pain, applied to healthy volunteers in experiment 1. The sensation was assigned to A_δ_ fibers when “pricking” but neither “dull” nor “pressing” were chosen, and to C fibers when the sum of the selections of “dull” or “pressing” was greater than that of the selection of “pricking”. In experiment 2, with an independent cohort, the three-descriptor questionnaire achieved sensitivity and specificity above 0.95 for distinguishing fiber preferential non-mechanical induced pain (laser heat, exciting A_δ_ fibers, and 5-Hz electric stimulation, exciting C fibers).

**Conclusion:**

A three-item verbal rating test using the words “pricking”, “dull”, and “pressing” may provide sufficient information to characterize a pain sensation evoked by a physical stimulus as transmitted via A_δ_ or via C fibers. It meets the criteria of a screening test by being easy to administer, taking little time, being comfortable in handling, and inexpensive while providing high specificity for relevant information.

## Introduction

In most medical settings, pain has a high prevalence with 61% in emergency medical care [1], almost 75% in patients reporting to general practice facilities [2], and with 12–80% chronic pain believed to affect the population [3]. It is therefore conceivable that the WHO advises for pain treatment as one of the major medical challenges. This is reflected by a broad research activity leading to a growing understanding of the pathophysiology of different pain syndromes. A_δ_ and C fibers, as the major pain-conducting nerve fiber systems, are involved to a different extent in these syndromes. For example, central sensitization to sensory input from A_δ_ fibers likely explains the response observed in the secondary zone of hyperalgesia [4], whereas in postherpetic neuralgia both fiber types are affected [5]. The fiber systems also show differences in their responsiveness to analgesics like opioids, which attenuate noxious C fiber input more potently than noxious A_δ_ fiber input [6], [7]. Fiber specificity also plays an important role in experimental pain. For example, punctate and blunt pressure stimuli produce pain transmitted via small myelinated A_δ_ and non-myelinated C fibers respectively [4], [8], [9], [10]. With the help of these specific stimuli, typically occurring pain qualities can be studied.

The differentiation of the nerve fiber systems transmitting these pain sensations is possible most sensitively by invasive methods such as microneurography. However, these sophisticated tests often cannot be carried out. A quick test providing fiber discrimination in experiments or at the patient bed to assess nerve fiber involvement in painful diseases is not available. Therefore, the objective of this study was to create a valid pain questionnaire that allows to discriminate quickly between pain transmitted via A_δ_ fibers and pain transmitted via C fibers. The development of the test originated from the McGill pain questionnaire [11], as it provided a large and well established set of verbal descriptors of the pain sensation to choose. Mechanical and non-mechanical induced pain stimuli were applied to healthy volunteers, who chose the descriptors that best matched their sensations.

## Methods

### Study design

In a single-blinded study design pain stimuli were applied by a single investigator on the arm or hand of the subjects, who were comfortably seated behind a black curtain shielding the subjects' view on the stimulus application. Subjects were informed about the purpose of the study. The questionnaire was developed from the 77 descriptors (sensory, affective and evaluative) of the validated German version of the McGill pain questionnaire [12]. The descriptors were presented in a fully randomized order, irrespective of their original category. Eight different versions of the questionnaire (i.e. randomizations) were used. In a first study (experiment 1) on 20 healthy volunteers of both sexes (medical students, aged 19 to 32 years, mean 24±3 years) punctate and blunt pressure stimuli were applied to evoke A_δ_ or C fiber mediated pain respectively [8]. The application order for punctate and blunt stimuli was randomized. Both stimuli were applied in the same session. After the application of each stimulus the subjects were asked to choose any number of descriptors from the questionnaire that described the pain sensation, they had experienced. The rating was carried out twice per stimulus.

In a second study (experiment 2), descriptors identified to distinguish best between A_δ_ and C fiber mediated pain were assessed with the the ratings of a new cohort of 20 healthy men and women (aged 21 to 33 years, mean 24±2.3 years) receiving noxious laser heat and electrical stimuli, known to excite preferentially A_δ_ [13] or C fibers [10] respectively. The procedure of stimulus application and rating for the second group was the same as for the first group.

The subjects' actual health had been checked by medical questioning. Medications except contraceptives were prohibited for one week, and alcohol for 24 h before the assessments. The study was conducted following the Declaration of Helsinki on Biomedical Research Involving Human Subjects. The University of Frankfurt Medical Faculty Ethics Review Board approved the study protocol. Informed written consent was obtained from all subjects.

### Pain stimuli

#### Mechanical stimuli

Neurophysiological and psychophysical studies in humans suggest that pain evoked by applying punctate and blunt stimuli is transmitted via small myelinated A_δ_ and unmyelinated C fibers respectively [4], [8], [9], [10]. Punctate pressure was produced by placing von Frey hairs of different strengths (4, 6, 8, 10, 15, 26, 60, 100, 180, 300 g; North Coast Medical Inc., Morgan Hill, CA, USA) perpendicularly onto the dorsal mid-phalanx of the right middle finger and increasing pressure until they bent slightly. Blunt pressure stimuli were applied using a pressure algometer with a circular and flat probe of 1 cm diameter (Commander Algometer, JTECH Medical, Midvale, Utah, USA; maximum pressure 111.6 N/cm^2^). It was placed perpendicularly onto the dorsal side of mid-phalanx of the right middle finger. The pressure was increased manually by the operator at a rate of approximately 9 N/cm^2^ per second until the desired pressure was obtained.

#### Thermal stimuli

Infrared Laser stimuli were administered at the back of the left hand using a thulium solid-state laser (Themis®, StarMedTec GmbH, Starnberg, Germany) at a wavelength of 1.96 µm. The stimuli were short (1 ms) pulses with a power of 150–600 mJ and a beam diameter of 5 mm. The high power of a laser stimulus produces a very fast heat ramp, which generally activates the terminals of both A_δ_ and C fibers [13], [14]. However, since A_δ_ fiber elicited first pain precedes the C fiber elicited second pain due to the different conduction velocities of the two afferents (∼10 m/s for A_δ_ and ∼1 m/s for C fibers), it can be easily distinguished when the stimulus is applied at a remote location such as the back of the hand [15]. Furthermore, “first pain” is more salient than “second pain” [16]. Therefore, in the present study, subjects were asked to rate and choose descriptors for the first sensation they experienced when being stimulated by the laser, which set the focus on the A_δ_ component.

#### Electrical stimuli

Electrical stimuli excite smaller fibers at lower frequencies because the maximum firing frequency is lowest for these fibers [17]. Nerve conduction studies and comparative quantitative sensory tests in patients with a sensory deficit, and pharmacological studies in animals and humans add indirect evidence that C fiber input is predominant for sensations evoked with 5 Hz sine waves [18], [19], [20], [21], [22], while higher frequencies (∼200 Hz) mainly activate A_δ_ fibers [23]. The 5-Hz sine wave electrical stimuli were applied via two gold electrodes placed on the medial and lateral side of the mid-phalanx of the left middle finger by means of a constant current device (Neurometer® CPT, Neurotron Inc., Baltimore, MD, USA; maximum output 20 mA).

#### Stimulus application procedure

Stimuli were administered in duplicates at intervals of approximately 30 s. As different quantitative stimulus intensities may confound the ratings of different stimulus qualities, all pain stimuli were applied at an equal intensity rated of (67 mm on a 100 mm visual analog scale VAS, ranging from 0 = no pain to 100 = maximum pain) by the respective subject. Two thirds (67/100) of maximum pain ensured that the stimuli were clearly painful and avoided that measurements were undertaken close to the maximum of the scale. The strength evoking this pain was calculated from the individual relationship between the pain intensity and the physical strength of the stimulus. This was established by fitting a power model (y = a+b·x^c^) to data obtained after administration of 10 mechanical stimuli at 10 different strengths (each von Frey hair applied once at a random succession or blunt pressure at 2, 5, 8, 10, 12, 15, 17, 20, 23 and 25 N/cm^2^, also applied at random order) immediately before the actual rating task. Specifically, for each stimulus quality, a parameter set was obtained consisting of 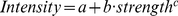. Obtaining, for example, the pressure needed to evoke a particular intensity, such as 67 mm VAS, requires rearranging the equation to 
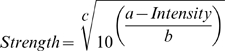
. With von Frey hairs, the one closest to the calculated strength was taken. For thermal (350, 400, 450, 500 and 550 mJ) and electrical stimuli (1.5, 2, 2.5, 3 and 3.5 mA), five stimuli were found sufficient for calibration of the stimulus strength needed to evoke a 67 mm VAS pain.

### Statistics

The similarity in pain intensities across stimuli was assessed by means of analysis of variance. For each verbal descriptor of the pain stimuli, the odds ratio of the number of its selections for C fiber mediated pain (pooled ratings of blunt mechanical pressure and 5-Hz electrical stimuli) and the number of its selections for A_δ_ mediated pain (pooled ratings of punctate mechanical pressure and laser heat) was calculated separately for each descriptor as (A · D)/(B · C), where the capital letters have the following meaning. A: number of selections of the descriptor for C fiber pain. B: total number of presentations of C fiber pain minus A. C: number of selection of the descriptor for A_δ_ fiber pain. D: total number of presentations of A_δ_ pain minus C. Descriptors of stimuli distinguishing between A_δ_ and C fiber mediated pain were identified by submitting the averages of the two verbal ratings of each mechanical stimulus to discriminant analysis. Variables were chosen stepwise for entry into the analysis by how much they lowered Wilk's lambda using F-statistics as the statistical criterion at an α level set at 0.05 (PASW statistics 18.02 for Linux, SPSS Inc., Chicago, IL, USA). The goodness of the discriminant function was estimated by leave-one-out cross-validated classification. Subsequently, the positive and negative predictive values, PPV and NPV respectively [24], of the identified set of verbal descriptors to distinguish A_δ_ and C fiber mediated pain sensations were assessed using the ratings of laser heat and 5-Hz electrical stimulation. This also served to calculate test sensitivity and specificity using standard equations [25].

## Results

Due to technical problems, experiment 2 failed to deliver data in one subject and results are therefore reported from n = 19 subjects. Punctate pressure (2.2±1.9 log g von Frey hairs), blunt pressure (19.4±4.6 N/cm^2^), laser heat (590.5±45.3 mJ) and electrical stimuli (3±0.78 mA) evoked pain at mean intensities of 64.2±15.8, 66.2±16.4, 54.2±18.7 and 68.8±16.4 mm VAS respectively (p>0.05).

**Figure 1:**
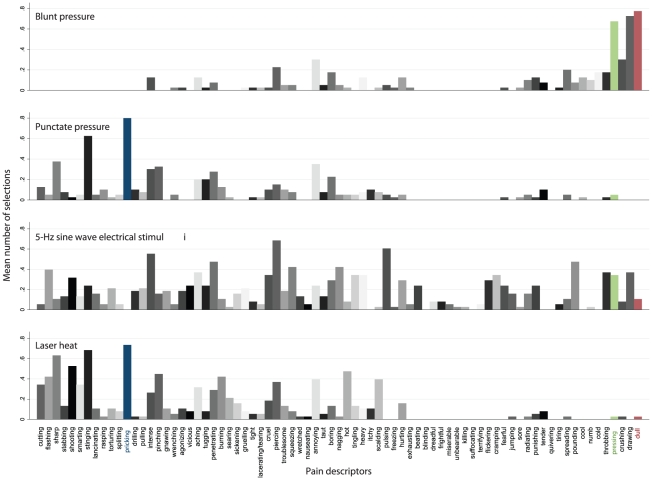
Choices of descriptors of the pain stimuli, summed across 20 subjects (A_δ_ selectivity more to the left, C fiber selectivity right). The colored bars indicate the three discriminators found to provide a questionnaire that distinguishes between A_δ_ and C fiber mediated pain (blue = “pricking”, green = “pressing”, red = “dull”, as specified in Figure 2).

The subjects used 2–70 descriptors for the stimuli, in median 9, 8.5, 16 and 24 descriptors (Figure 1) for punctate pressure, blunt pressure, laser heat and electrical stimuli (p<0.001), without substantial differences between first and second ratings in the repetitions (e.g., in median 4.5 items for both ratings of punctuate pain). Items most frequently chosen for punctate pressure pain were “pricking” (32 hits), “stinging” (25 hits), and “sharp” (15 hits), while “dull” (31 hits), “pressing” (27 hits), and “squeezing” (29 hits) were the most frequent descriptors for pain from blunt pressure.

**Figure 2:**
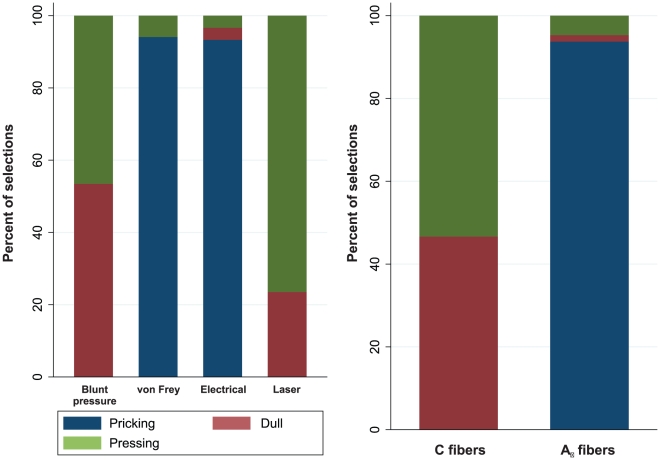
Cumulative presentation of the selections of the three discriminators found to provide a questionnaire that distinguishes between A_δ_ and C fiber mediated pain. Left: presentation separately for the four pain models and standardized at 100% of the ratings for each model. Right: presentation of the selections of the three discriminators, pooled for predominately A_δ_ fiber mediated pain sensations evoked with von Frey hair puntuate pressure stimuli or with laser heat stimuli and C fiber mediated pain sensations evoked with blunt pressure stimuli or with 5-Hz electrical stimuli.

Discriminant analysis of the ratings from experiment 1 identified “pricking”, “dull” and “pressing” as distinguishing best between A_δ_ mediated (punctate pressure) and C fiber mediated (blunt pressure) pain sensations (Figure 2). With these three descriptors, 95% of cross-validated cases were correctly classified. The sensation was assigned to A_δ_ fibers when “pricking” was chosen but neither “dull” nor “pressing”. In contrast, classification was toward C fibers when the sum of the selections of “dull” or “pressing” was greater than that of the selection of “pricking”. The subject falsely classified on the basis of these descriptors had not chosen “pricking” for the description of the punctate stimulus, which caused wrong assignment of A_δ_ pain, while the single selection of “dull” for pressure pain triggered correct assignment of C fiber pain.

**Figure 3:**
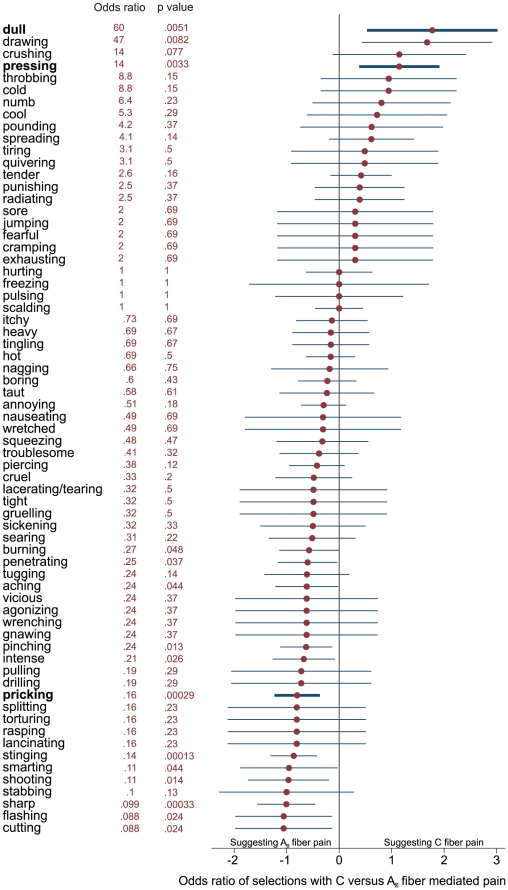
Odds ratios. Odds ratios, calculated as (A · D)/(B · C), where A is the number of selections of the descriptor for C fiber pain, B the total number of presentations of C- fiber pain minus A, C the number of selection of the descriptor for A_δ_ fiber pain, and D the total number of presentations of A_δ_ pain minus C, and 95% confidence intervals, calculated as odds ratio±1.96 · (1/A+1/B+1/C+1/D)^0.5^, of the selections of the descriptors for C fiber mediated pain (pooled ratings of blunt mechanical pressure and 5-Hz electrical stimuli) and the number of selections of the descriptors for A_δ_ mediated pain (pooled ratings of punctate mechanical pressure and laser heat), sorted from top to bottom for decreasing selectivity for C fibers and increasing selectivity for A_δ_ fibers. The descriptors used in the questionnaire test (Figure 2) are marked in bold letters and thicker lines. Odds ratios and the corresponding p-values resulting from χ^2^ statistics are indicated at the right of the descriptors. Please note that the 10 descriptors that were never used are excluded and therefore, the total number of displayed descriptors is 67.

Applying this algorithm to the results of the cohort of experiment 2 (laser heat and electrical stimuli, n = 19) (Figure 3) resulted in 18 correct positive, 19 correct negative, one false negative and zero false positive diagnoses of A_δ_ fiber mediated pain, which corresponds to a PPV of 1, an NPV of 0.95, a test sensitivity of 0.95, and a specificity of 1. Correspondingly, for C fiber mediated pain zero false negative and one false positive diagnoses were obtained, which resulted in values of PPV, NPV, sensitivity and specificity of 0.95, 1, 1 and 0.95 respectively. In fact, the diagnoses could be equally correctly made by regarding only the presence or absence of “pricking” among the ratings of the stimuli. There were no subjects in the total study group using none of the three descriptors in any single pain estimate, which rules out the possibility of missing values in the analysis.

Odds ratios (and 95% confidence intervals) were calculated for the number of selections of the descriptors for C fiber mediated pain by pooling the ratings of blunt mechanical pressure and 5-Hz electrical stimuli. The same was done for the descriptors for A_δ_ mediated pain by pooling the ratings of punctate mechanical pressure and laser heat (Figure 3).

Besides “pricking”, “dull” and “pressing”, a few alternative descriptors distinguished between fiber predominance almost as well and could be contemplated as alternatives, such as “sharp” and “stinging” clearly pointing at A_δ_ and “dull”, “drawing” and “pressing” indicating C fiber predominance (Figure 3). They were not included in the final discriminant function probably because they provided only redundant information to the included items, which is supported by values of Cohen's κ of >0.6 indicating substantial agreement [26] between these items and the corresponding selected descriptors.

## Discussion

The major finding of the present study was that three descriptors of pain can discriminate between pain sensation preferentially conducted by either A_δ_ or C nerve fibers with a specificity of 95%. This result has been obtained by analyzing the verbal ratings of mechanical stimuli and verifying the findings at non-mechanic stimuli rated by different subjects.

Although further analysis suggested that a rating of the pain stimulus as “pricking” or not can equally distinguish these fiber preferences, the three item test was preferred because it is known that the multiple-choice format provides a significantly more reliable measure than the true-false format [27]. Thus, possible instructions given to the subjects for the three-item test could be: “From the words ‘pricking’, ‘dull’, ‘pressing’, please choose any number of words that best describe the pain you experience.”

Whether a stimulus excites predominantly A_δ_ or C fibers is commonly asked in basic pain research. Functional magnetic resonance imaging results showed that the central processing of these two different pain stimuli involves different cortical areas [28]. A_δ_ and C fiber mediated pain both activated areas of the well-known nociceptive network (“pain matrix”) [29]. However, C fiber stimulation, when directly compared with A_δ_ fiber stimulation, additionally activated the frontal operculum and anterior insular cortex, which was interpreted as C fibers being engaged in homeostatic and interoceptive functions in another manner than A_δ_ fibers. High-frequency stimulation induced potentiation of pain evoked by electrical stimuli was described mainly with “hot” and “burning” or mainly with “piercing” and “stinging” depending on whether the facilitation affected either the conditioned cutaneous C-fiber or A_δ_ fiber pathway, which agrees well with the present results. Further supporting evidence for the presented finding that fiber selectivity can be identified using verbal descriptors is the dissimilarity of the sensory descriptor choices for tonic and phasic experimentally induced pain [30], which are also believed to affect predominantly one of the two fiber systems.

Another area, where the involvement of pain fiber types could not yet be fully clarified is visceral pain, where the exact mechanism of convergence of visceral and somatic afferents is still poorly understood [31]. Research in pain disorders, such as the complex regional pain syndrome, may also benefit from the results of our study, because the sensations characterizing this disease are usually described as “aching”, “burning” and “radiating” [32]. While “radiating” has a poor discriminative potential (Figure 3), “aching” and “burning” clearly point at an A_δ_ fiber origin of this disease. It is noteworthy that many common questionnaires for the screening of neuropathic pain include “burning” as a descriptor for C-fiber involvement [33]classification is not supported by our observations. Similar results were obtained by other groups, as well, when assessing neuropathic pain in cancer patients [34]. Although not a standard clinical practice yet, in the future a discrimination of involved fibers may help to identify the pain medication suited best for a particular condition. As a recent study on mirror visual feedback in the treatment of deafferentiation pain has shown that the pain-alleviating effects of a treatment can indeed be related to the different pain descriptors reported by the patients [35].

A special case are studies on the underlying mechanisms of acupuncture. Early experiments focused on A_δ_ fibers because the pin prick sensation often elicited on needle insertion [36]. Recent studies, however, emphasize the importance of the acupuncture-specific needling sensation “deqi” [37], [38] usually described with the words “soreness”, “aching”, “deep pressure”, “heaviness”, “fullness/distension”, “tingling”, “numbness”, “dull pain”, “warmth”, “cold” and “throbbing” [37], while “sharp pain” is usually considered not to be part of this sensation [38]. The present results support that the major part of the “deqi” sensation can be explained by C fiber stimulation, as most words clearly describe C fiber related sensations (“pressing”, “numb”, “dull”, “cold”, “throbbing”), while “aching” is the only descriptor more related to A_δ_ fibers. The other words were either not suitable for discrimination (“tingling”, “heaviness”) or not part of the McGill questionnaire (“fullness”, “warm”).

The present test fulfills the criteria for a rapid screening test in a clinical or experimental setting. It is easily comprehensible, takes little time to administer, is comfortable in handling, is inexpensive, and is reliable as it rarely produces false positive results. Reliability is important as a less specific test would lead to many follow-up investigations with more extensive pain tests. This is difficult to handle when nerve conduction preferences are not the primary focus of the experiment but may prove important for interpreting and discussing its results.

However, compared with more extensive non-invasive tests such as the McGill questionnaire [11] or even a quantitative sensory testing, a simple three-item questionnaire is unlikely to be as sensitive to sensory disturbances as a comprehensive test. Further limitations of the present test may reflect limitations of sensory tests in general. In a neuro-psychiatric setting, test performance is not independent of the subjects' cognitive function. Subjects with deteriorated cognitive function may provide less reliable responses than the present subjects. However, independence from cognitive performance cannot be achieved with psychophysical tests but requires more objective means such as microneurography. Nevertheless, the simplicity of this test makes it applicable in most pain experiments and may therefore provide information about the involved nerve fibers that otherwise had not been acquired at all. Another possible limitation is imposed by imperfect knowledge on the exact fiber recruitment by the different stimuli. Moreover, the 5 Hz sine wave electrical stimulation (C fiber predominance) seems to differ from the other sensations as it was rated with more descriptors over the whole range without a shift to the right side of the graph as seen for blunt pressure (Figure 3). This may hint at an induction of mechanical sensations and partial recruitment of A_δ_ fibers by electrical stimulation with 5 Hz pulses.

Results from this study suggest that a simple three-item verbal rating test using the words “pricking”, “dull”, and “pressing” may provide sufficient information to characterize a pain sensation evoked by a physical stimulus as transmitted via A_δ_ or via C fibers. It meets the criteria of a screening test by being easy to administer, taking little time, being comfortable in handling, and inexpensive while providing high specificity for relevant information. Apart from these practical aspects, however, it has to be acknowledged that this short test is limited to its purpose of quick fiber distinction in a pain context while it cannot replace a comprehensive phenotyping. It may nevertheless provide a major improvement of the experimental design when information about fiber preference is frequently not gathered at all.
